# Comparative effects of mechanical and functional alignment in bilateral robotic total knee arthroplasty: a randomized controlled trial

**DOI:** 10.1186/s42836-025-00310-5

**Published:** 2025-05-07

**Authors:** Thakrit Chompoosang, Utain Ketkaewsuwan, Patcharavit Ploynumpon

**Affiliations:** Department of Orthopedics, Rajavithi Hospital, Bangkok, 10400 Thailand

**Keywords:** Functional alignment, Mechanical alignment, Total knee arthroplasty, Robotic-assisted surgery, Soft tissue release

## Abstract

**Background:**

Functional alignment (FA) in total knee arthroplasty (TKA) can achieve soft tissue balance by fine-tuning adjustments of bony resections and component alignment with less soft tissue release. However, joint line orientation relative to the floor in the knee and ankle after TKA is not well studied.

**Methods:**

A randomized-controlled trial was performed in 30 patients with robotic-assisted bilateral TKA using FA and mechanical alignment (MA) in the same patient. The outcome measures were as follows: (1) standing radiographic knee and ankle alignment; (2) clinical outcomes at 1, 3 and 6 months postoperatively (including forgotten joint score (FJS), KOOS, knee range of motion); (3) patient satisfaction score; and (4) soft tissue release.

**Results:**

Postoperative hip-knee-ankle angles between the FA and MA groups were similar (2.4° versus 2.4°, *P* = 0.952). Knee joint line orientation was significantly more parallel to the floor in the FA group (3.0° versus 4.7°, *P* < 0.001). There was no significant difference in ankle joint line orientation relative to the floor in the FA and MA groups (91.0° versus 92.4°, *P* = 0.099 for tibial plafond inclination and 92.5° versus 93.2°, *P* = 0.564 for talar dome inclination). However, in knees with preoperative varus with apex distal joint line orientation (coronal plane alignment of the knee (CPAK) classification type I), FA significantly achieved a more parallel knee and ankle joint line orientation relative to the floor (3.1° versus 5.1°, *P* = 0.002 for knee and 91.0° versus 93.5°, *P* = 0.028 for tibial plafond inclination). FA can obtain a balanced knee with significantly lower posteromedial releases (23.3% versus 76.7%, *P* < 0.001), with no superficial MCL release needed (0% versus 6.67%, *P* < 0.01). The FA group achieved significantly higher FJS at 3 months (53.3 versus 46.0, *P* = 0.015) and 6 months (67.8 versus 57.8, *P* < 0.001) with a higher patient satisfaction score (79.2 versus 84.3, *P* = 0.001).

**Conclusion:**

Functional alignment can control the overall lower limb alignment similarly to mechanical alignment, with a knee joint line more parallel to the floor. Additionally, the ankle joint line was more parallel in knees with CPAK type I. FA can also provide a more balanced knee with less soft tissue release, a higher functional score, and greater patient satisfaction compared to mechanical alignment.

## Introduction

Total knee arthroplasty (TKA) is a highly effective operation and is widely used for the treatment of patients with symptomatic knee osteoarthritis. Mechanically aligned TKA (MA-TKA), the most widely used alignment strategy, aims to position femoral and tibial components perpendicular to the mechanical axis and has demonstrated good long-term implant survival [1]; however, approximately 20% of patients [2, 3] have been found to express dissatisfaction following primary TKA. To improve patient satisfaction, many alternative knee alignment philosophies have been described.

A study [4] of healthy individuals found that when the knee joint line is parallel to the floor in a bipedal stance, constitutional varus does not affect this joint line orientation. In contrast, for symptomatic arthritic patients with varus alignment, the knee joint line slants down to the lateral side. Kinematically aligned TKA (KA-TKA), which aims to restore the native pre-arthritis knee, has been found to achieve knee joint line orientation more parallel to the ground than that of mechanically aligned TKA (MA-TKA) [5].

Currently, TKA evaluation is focused mainly on knee alignment, and the pathological findings and other adaptive changes in the ankles are usually neglected. A recent study [6] reported that the restoration of ankle joint line orientation after KA-TKA was more horizontal to the floor and closer to that of native ankle joints than after MA-TKA.

With the advent of computer-assisted surgery (CAS), the use of navigated or robotic, functionally aligned TKA (FA-TKA) has been developed. FA-TKA aims to restore native joint line height and obliquity while achieving ligament balance by fine-tuning adjustments of bony resections and component position [7]. To the best of our knowledge, there has been no detailed report regarding the effects of FA-TKA on ankle alignment and knee joint line orientation relative to the floor.

Our study aimed to compare the postoperative effects of MA-TKA and FA-TKA on knee joint line orientation, ankle alignment, and clinical outcomes.

## Patients and methods

In our study, clinical outcomes using the clinical score were used as the primary outcome measure to compare between groups. The restoration concept of natural knee kinematics in FA-TKA could result in less tension on the soft tissues, leading to a reduced need for soft tissue release. This may contribute to differences in functional outcomes between the two groups.

Radiological outcomes are also used as a second outcome due to differences in surgical concept between groups.

Ethical approval was obtained from the institutional ethical review board, and the trial was registered with ClinicalTrials.gov, NCT06259032. All participants gave written informed consent before participating. Inclusion criteria consisted of patients with symptomatic bilateral knee osteoarthritis who required primary TKA, were willing to undergo bilateral simultaneous TKA, and were able to give informed consent. Exclusion criteria consisted of patients with knee ligament deficiency requiring constrained prosthesis; bone loss with augmentation need; history of fracture around the knee or previous osteotomy; history of ankle fracture, neuromuscular disorder, or movement disorder; and inability to attend the study follow-up program for at least 3 months postoperatively. All participants gave informed consent. The right knee was randomized to either FA or MA using a computer-generated block of four randomizations and opaque sealed envelopes. The left knee was assigned to the alternative technique, with the patient blinded to the alignment strategy. During surgery, the sealed envelopes were opened by the operating room staff to inform the surgeon about the intervention side, and the surgeon will always start the surgery at the right knee first, regardless of whether it is FA or MA.

Pre-operative demographic data of age, gender, and body mass index (BMI) were recorded for all patients. Radiographic knee parameters included coronal plane alignment of the knee (CPAK) [8] classification; hip-knee-ankle (HKA) angles; lateral distal femoral angle (LDFA); medial proximal tibia angle (MPTA); knee joint line orientation (KJLO); and radiographic ankle parameters, consisting of tibial plafond inclination, talar inclination and tibiotalar tilt angle. Clinical scores, including forgotten joint score (FJS) [9], Knee Injury and Osteoarthritis Outcome Score (KOOS) [10], and knee range of motion were recorded in all cases.

Radiographic parameters were assessed with closed-leg standing long-leg radiographs. For AP radiograph, foot orientation angle and equal distance between both feet (in contact) were set, which is standardized by using a foot template, with the patella facing forward, which is confirmed by preview digital x-ray films. Radiographic images were digitally acquired using a picture archiving and communication system (PACS), and all measurements were taken using Synapse-PACS software (Fujifilm, Tokyo, Japan). Two orthopedic surgeons who were not involved in this study independently performed all the radiographic assessments to evaluate interobserver reliability. One rater remeasured the selected radiographs within a 2-week interval. Intraclass correlation coefficients of intra- and inter-observer reliabilities were excellent (> 0.85; range, 0.85–0.99). The final analysis was performed with the measurements taken by one rater.

**Fig. 1:**
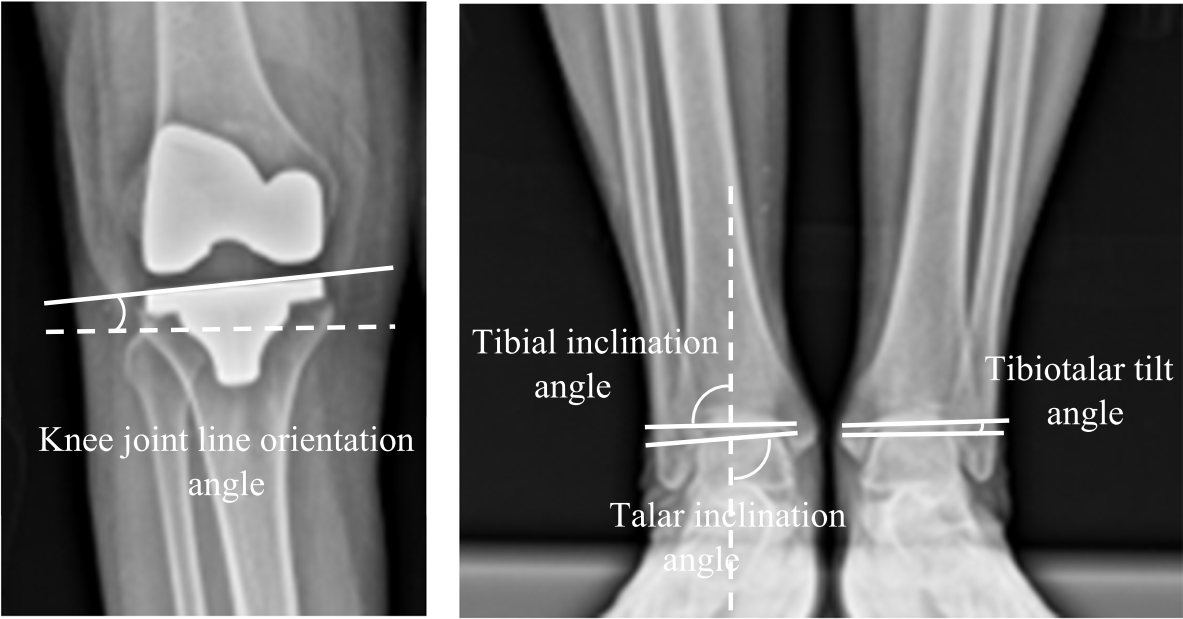
Radiographic measurements: (a) knee joint line orientation (KJLO) angle, which had a positive value when the joint line was slanted down to the lateral side; (b) Tibial inclination, talar inclination, and tibiotalar tilt angle

KJLO was defined as the angle formed between the tibial joint line and a line parallel to the floor and had a positive value when the joint line was slanted down to the lateral side. The HKA angle was defined as the angle between the mechanical axis of the femur and tibia and was described as a deviation from 180 degrees. Varus’ overall limb alignment was expressed as a positive value. LDFA described the lateral angle formed between the femoral mechanical axis and the joint line of the distal femur, and MPTA was defined as the medial angle formed between the tibial mechanical axis and the joint line of the proximal tibia. Tibial plafond inclination was the lateral angle between the distal tibial articular surface and a vertical line to the ground. Talar inclination was defined as the medial angle between the talar dome and a vertical line to the ground. Tibiotalar tilt angle was the angle between the distal tibia articular surface and the talar dome and had positive values when the angle was open on the lateral side (Fig. 1).

Intraoperative outcomes consisted of operative time, defined by “skin-to-skin” (incision until final stitch complete), and requirement of soft tissue release. Pain scores described by the visual analog scale (VAS) were recorded daily for the first 3 days after the operation. Knee range of motion (ROM) was recorded at postoperative day 3, 1 month, 2 months, 3 months, and 6 months postoperatively, while radiographic outcomes were measured at 3 months postoperatively. FJS and KOOS scores were recorded at 1 month, 2 months, 3 months, and 6 months postoperatively. Patient satisfaction score was measured at 3 and 6 months postoperatively, and they were recorded as the patient’s self-rated satisfaction on a VAS from 0 (very unsatisfied) to 100 (very satisfied). After removing the patient identifier, all clinical outcomes were sealed, collected, and analyzed by an experienced outcome assessor who was not involved in the study.

All knee operations were performed using the Mako robotic-arm assisted system (Stryker, USA) with cemented Cruciate retaining, fixed-bearing implant (Triathlon; Stryker, USA) without patellar resurfacing. All procedures were carried out by a single senior staff surgeon (T.C.).

### Surgical technique

All patients were evaluated with a preoperative computer tomography (CT) scan of the lower limb, which generated a three-dimensional model, and preoperative plans were created by Mako software.

The MA group was assigned to have neutral alignment with femoral and tibial components perpendicular to their respective mechanical axes. Femoral rotation was set perpendicular to the transepicondylar axis (TEA). Tibial posterior slope was set between 0 and 3 degrees. Soft tissue releases were done using selective medial soft tissue release. Starting with the deep medial collateral ligament (dMCL), then the superficial medial collateral ligament (sMCL) or posterior oblique ligament (POL) until soft tissue balancing was achieved [11].

In the FA group, the implant position was based on equal bony resection depths set to 6.5 mm on both medial and lateral sides at the most distal and posterior point of the femoral condyles. Proximal tibial cut was set to 7 mm equal resections medially and laterally. All bone resection depths were adjusted for bone loss as described by Howell [12]. The tibial posterior slope was set according to the native slope. The implant position and resection depths were adjusted to achieve a balanced knee (target virtual gap was set to 20 mm with a difference in gap of 2 mm or less), by balancing extension gaps with coronal adjustment of the more diseased side first (usually tibial component first for a varus knee). Overall boundaries were as follows: HKA within 3 degrees of neutral alignment, femoral coronal alignment between 6 degrees valgus and 3 degrees varus, femoral rotation from posterior condylar axis (PCA) to 5 degrees external rotation to TEA, tibial coronal alignment between 6 degrees varus and 3 degrees valgus and posterior tibial slope between 0 and 3 degrees. If, after all adjustments, the balanced gap could not be achieved, minimal selective releases of the tight structures were performed by the surgeon using the needle puncture technique.

After all bone cuts had been executed, trial implants were inserted, and maximal gaps were then obtained with trial components in place. If the knee was imbalanced, soft tissue release was performed.

### Post-operative care

Both groups received the same postoperative pain management protocol (comprising Naproxen 250 mg oral twice daily, Paracetamol 500 mg q 4 h, Tizanidine (4 mg) 2 oral q 6 h and Gabapentin (100 mg q 8 h), all of the patients were allowed to ambulate the day after the surgery by experienced physiologists who use same standard post-operative rehabitation protocol.

### Statistical analysis

Data normality was confirmed using the Shapiro–Wilk test. Continuous variables were analyzed with the paired t-test and independent samples t-test for parametric data, while the Wilcoxon matched pairs signed rank test was employed for nonparametric data. Categorical data were compared with the chi-square test and Fisher’s exact test, and frequency distributions were compared with the 2-sample Kolmogorov–Smirnov test. All dependent measurements were reported as mean ± standard deviation, and statistical significance was defined as *P* < 0.05.

### Power analysis

Power calculations, based on the study of Dossett et al. (2012) [13] and using the standard formula [14], indicate that a sample size of 18 knees per group will provide a minimum of 80% power to detect significant effects at *P* < 0.05. To account for potential dropouts, a minimum of 20 knees per group will be included to maintain the desired power.

The Minimal Clinically Important Difference (MCID) for clinical outcomes differences between groups, using the KOOS score [15], was set at 9 [16]. The effect size of clinical outcomes was also calculated using Cohen’s d formula.

## Results

### Baseline data

**Fig. 2:**
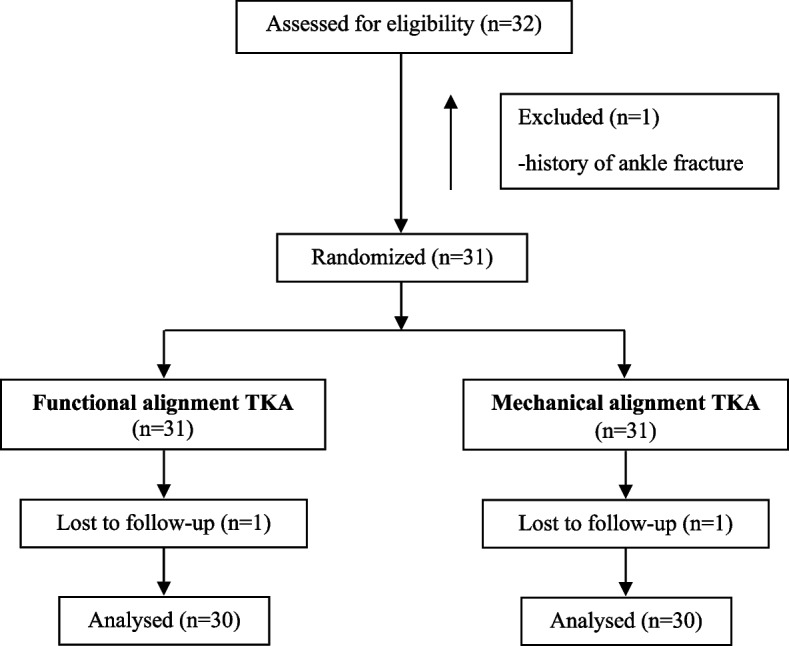
CONSORT flow diagram

**Fig. 3:**
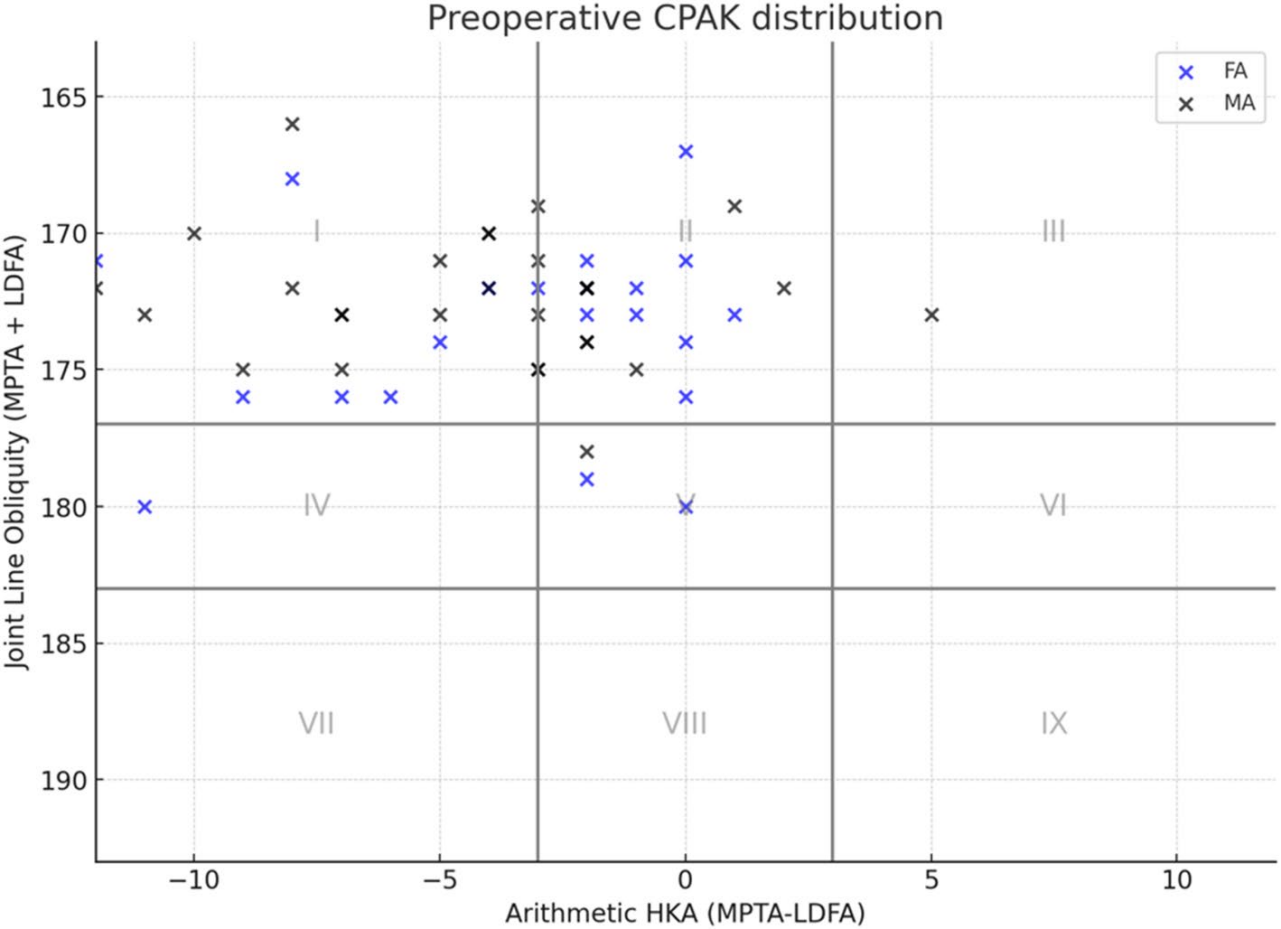
Distribution of arithmetic hip-knee-ankle angle (aHKA) and joint line obliquity (JLO) by Coronal Plane Alignment of the Knee (CPAK)

Between November 2023 and February 2024, 32 patients were enrolled in the study. Two were later excluded due to a history of ankle fracture with plate fixation (*n* = 1) and being lost to follow-up before 3 months (*n* = 1). A CONSORT flow diagram is shown in Fig. 2. The final 30 patients were analyzed, with a mean age of 67.9 years (SD ± 6.5) and a mean BMI of 27.7 kg/m^2^ (SD ± 5.0). Twenty-six were female (86.7%). Preoperative CPAK distributions are demonstrated in Table 1. Thirty-three knees (55%) had constitutional varus with apex distal joint line orientation or CPAK type I, followed by 21 knees (35%) with neutral alignment and distal joint line orientation. Preoperative data are illustrated in Fig. 3 and detailed in Table 2. Clinical scores were comparable in the two groups with no statistically significant differences, and radiographic parameters also showed no significant difference.

**Table 1:** Preoperative CPAK distributions

	Total	FA	MA	*p* value
n	60	30	30	
Preoperative CPAK, n (%)				0.225
I	33 (55)	13 (43.3)	20 (66.7)	
II	21 (35)	13 (43.3)	8 (26.7)	
III	1 (1.7)	0	1 (3.3)	
IV	1 (1.7)	1 (3.3)	0	
V	4 (6.7)	3 (10)	1 (3.3)	
VI	0	0	0	
VII	0	0	0	
VIII	0	0	0	
IX	0	0	0

**Table 2:** Preoperative data

Preoperative measurements (˚)	FA (n = 30)	MA (n = 30)	p-value	Mean diff	95%CI
	Mean (SD)	Mean (SD)			
KOOS	36.8 (12.6)	34.5 (15.1)	0.526	2.3	(− 5.04, 9.64)
FJS	26.9 (18.0)	24.7 (15.4)	0.598	2.2	(− 6.65, 11.05)
Flexion range	121.2 (10.0)	122.4 (7.3)	0.599	− 1.2	(− 5.82, 3.42)
HKA	8.8 (5.4)	10.3 (7.0)	0.379	− 1.5	(− 4.8, 1.8)
LDFA	88.7 (2.2)	88.4 (2.3)	0.649	0.3	(− 0.89, 1.49)
MPTA	85.0 (3.0)	84.0 (2.4)	0.138	1.0	(− 0.43, 2.43)
KJLO	3.6 (1.4)	3.0 (1.3)	0.090	0.6	(− 0.11, 1.31)
Tibial plafond inclination	95.5 (5.5)	96.7 (5.3)	0.378	− 1.2	(− 4.05, 1.65)
Talar inclination	97.5 (5.4)	98.0 (5.3)	0.737	− 0.5	(− 3.33, 2.33)
Tibiotalar tilt angle	2.2 (2.1)	2.1 (2.0)	0.901	0.1	(− 0.98, 1.18)

### Intraoperative outcomes

Additional soft tissue release was significantly lower in the FA group, with 7 knees (23.3%) in the FA group needing posteromedial releases compared with 23 knees (76.7%) in the MA group (*P* < 0.001. No MCL release was needed in the FA group compared with 2 knees (6.67%) in the MA group. Operative time in the FA and the MA groups was comparable (63.4 versus 66.2 min, respectively, *P* = 0.623).

### Radiographic outcomes

Postoperative HKA angles were very similar in the two groups (2.4° in both groups, *P* = 0.952) (Table 3). The change in postoperative KJLO was significantly different: it decreased in the FA group (mean decrease 0.6°), while in the MA group it increased (mean increase 1.7°, *P* < 0.001). The final postoperative KJLO was more parallel to the floor in the FA group (3.0° versus 4.7° in their MA counterparts, *P* < 0.001). Tibial plafond inclination and talar inclination were significantly lower postoperatively (*P* < 0.001) and were equally parallel to the floor in both groups, with no significant difference between them. Tibiotalar tilt angle improved significantly in both groups, with no significant difference.

**Table 3:** Radiographic outcomes

Radiographic parameters (˚)	FA (n = 30)	MA (n = 30)	p-value	Mean diff	95%CI
	Mean (SD)	Mean (SD)			
HKA	2.4 (2.1)	2.4 (2.1)	0.952	0.0	(− 2.57, − 0.83)
LDFA	89.5 (2.7)	90.7 (2.5)	0.092	− 1.2	(− 3.29, − 1.31)
MPTA	88.8 (2.1)	89.6 (1.5)	0.096	− 0.8	(− 3.09, 0.29)
KJLO	3.0 (1.8)	4.7 (1.5)	< 0.001	− 1.7	(− 2.57, − 0.83)
Δ KJLO	− 0.6 (2.2)	1.7 (1.5)	< 0.001	− 2.3	(− 3.29, − 1.31)
Tibial plafond inclination	91.0 (3.1)	92.4 (3.3)	0.099	− 1.4	(− 2.35, 1.95)
Δ Tibial plafond inclination	− 4.5 (3.9)	− 4.4 (3.9)	0.870	− 0.1	(− 1.14, 0.34)
Talar inclination	92.5 (4.1)	93.2 (4.3)	0.564	− 0.7	(− 1.22, 0.22)
Δ Talar inclination	− 5.0 (4.4)	− 4.8 (3.7)	0.874	− 0.2	(− 2.57, − 0.83)
Tibiotalar tilt angle	1.1 (1.4)	1.5 (1.4)	0.272	− 0.4	(− 3.29, − 1.31)
Δ Tibiotalar tilt angle	− 1.1 (1.2)	− 0.6 (1.5)	0.198	− 0.5	(− 3.09, 0.29)

**Fig. 4:**
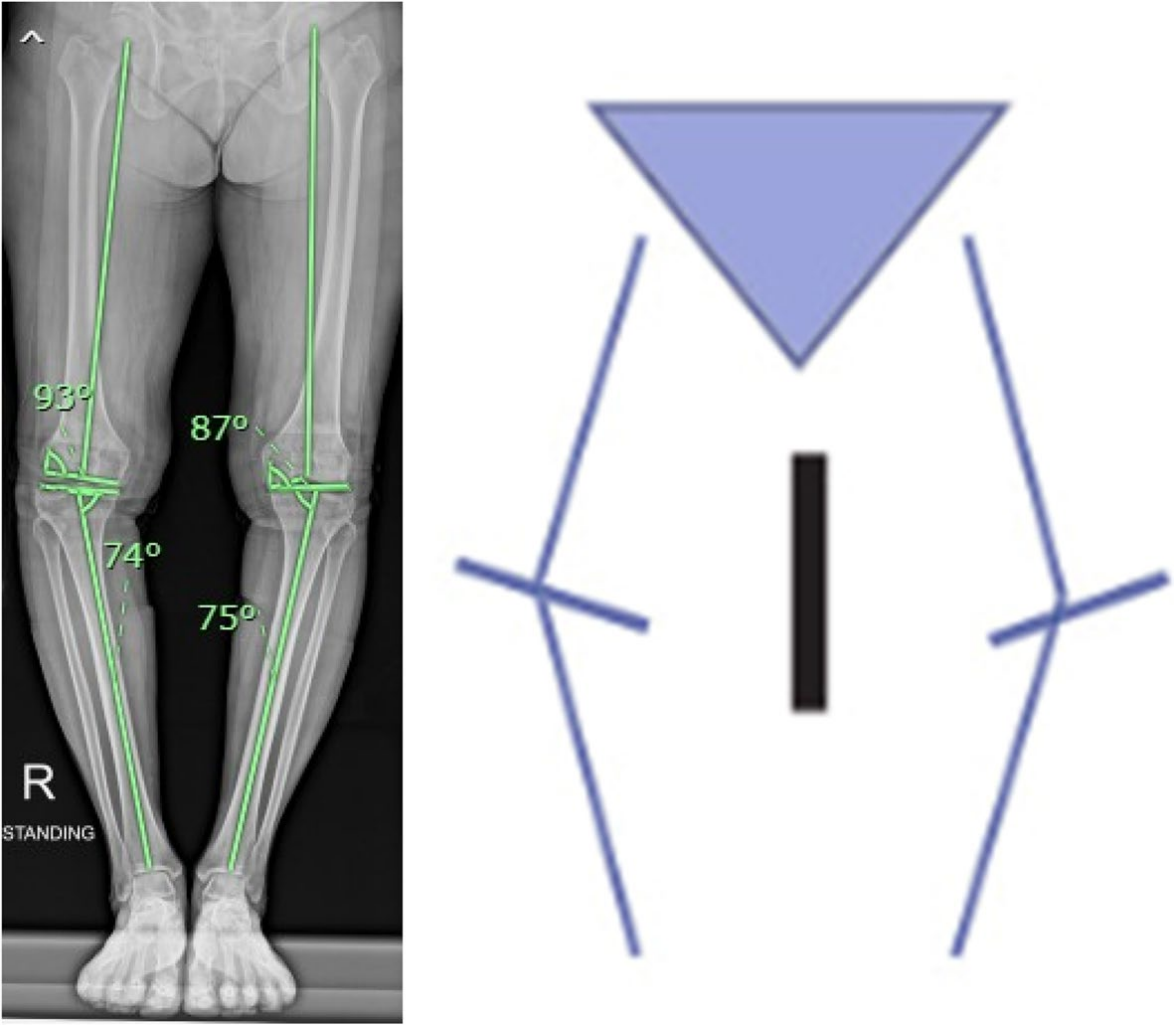
Example of CPAK 1

**Fig. 5:**
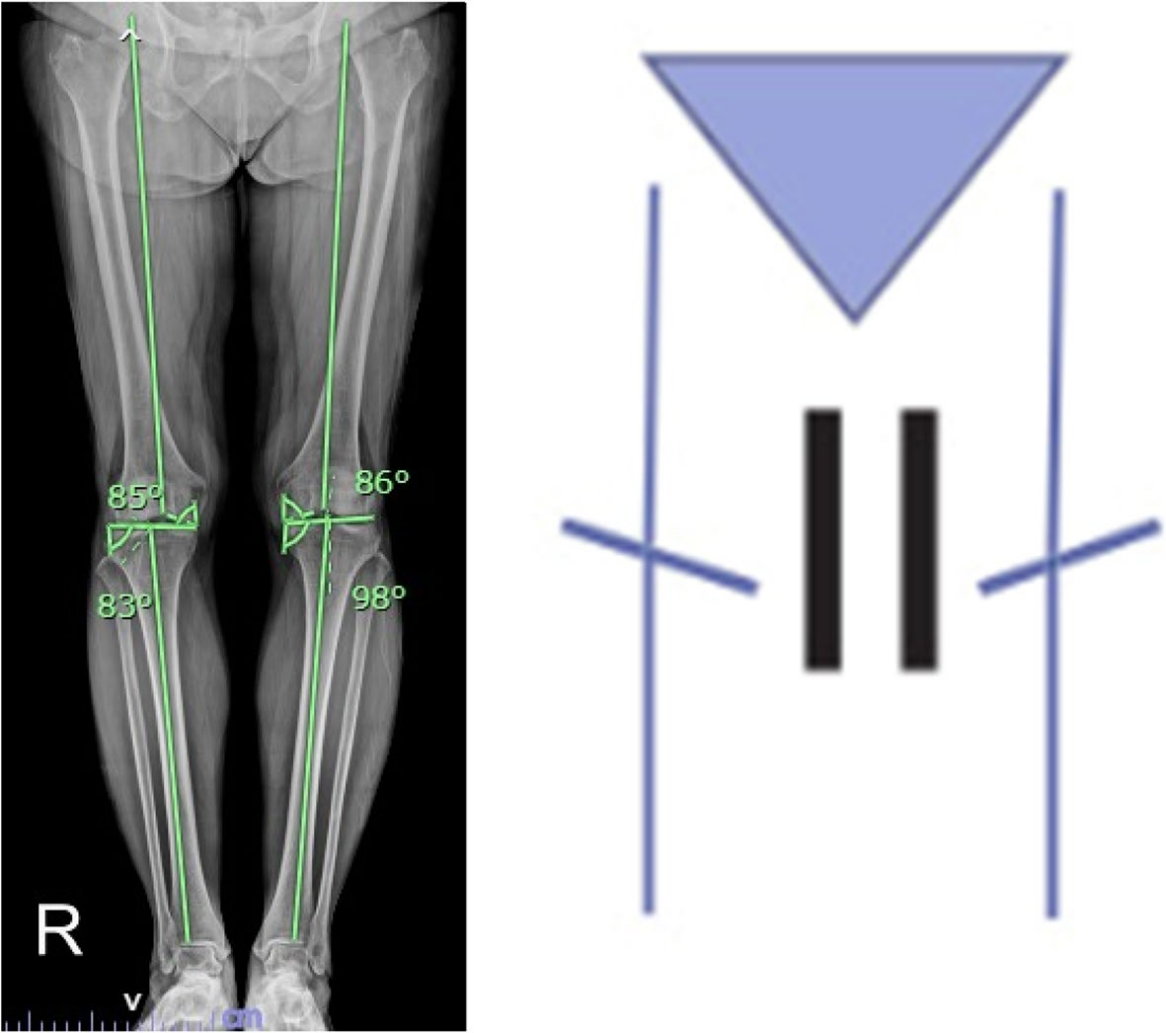
Example of CPAK 2

With regard to the CPAK phenotypes (Table 4) (Figs. 4 and 5), the change in postoperative KJLO in the FA group was significantly different from that of the MA group (decrease of 0.5° versus increase of 2.0°, *P* < 0.001) and KJLO in the FA group was more parallel to the floor in CPAK type I (3.1° versus 5.1° respectively, *P* = 0.002). There were no statistically significant differences between postoperative ankle parameter changes and the final ankle parameters in the two groups, except that tibial plafond inclination in the FA group was more parallel to the floor in CPAK type I (91.0° versus 93.5°, *P* = 0.028).

**Table 4:** Radiographic outcomes CPAK type 1 and 2 subgroups

CPAK	Radiographic parameters (˚)	FA	MA	p-value	Mean diff	95%CI
		Mean (SD)	Mean (SD)			
I FA *n* = 13 MA *n* = 20	Preoperative					
	HKA	11.4 (6.0)	13.5 (5.4)	0.309	− 2.1	(− 5.11, 0.91)
	LDFA	89.5 (1.9)	89.3 (2.0)	0.687	0.2	(− 0.83, 1.23)
	MPTA	82.4 (1.7)	82.8 (2.0)	0.537	− 0.4	(− 1.38, 0.58)
	KJLO	3.6 (1.5)	3.2 (1.3)	0.361	0.4	(− 0.34, 1.14)
	Tibial plafond inclination	96.8 (4.1)	98.5 (4.5)	0.275	− 1.7	(− 3.97, 0.57)
	Talar inclination	99.3 (5.1)	100.0 (4.0)	0.668	− 0.7	(− 3.12, 1.72)
	Tibiotalar tilt angle	2.9 (2.6)	2.5 (2.4)	0.655	0.4	(− 0.92, 1.72)
	Postoperative					
	HKA	2.8 (2.7)	2.9 (2.1)	0.309	− 0.1	(− 1.38, 1.18)
	LDFA	89.2 (2.6)	90.8 (2.9)	0.122	− 1.6	(− 3.05, − 0.15)
	MPTA	88.4 (2.6)	89.5 (1.5)	0.196	− 1.1	(− 2.22, 0.02)
	KJLO	3.1 (2.0)	5.1 (1.3)	0.002	− 2.0	(− 2.89, − 1.11)
	Δ KJLO	− 0.5 (1.9)	2.0 (1.4)	< 0.001	− 2.5	(− 3.38, − 1.62)
	Tibial plafond inclination	91.0 (3.0)	93.5 (4.5)	0.028	− 2.5	(− 4.52, − 0.48)
	Δ Tibial plafond inclination	− 5.8 (3.5)	− 5.1 (3.9)	0.593	− 0.7	(− 2.66, 1.26)
	Talar inclination	93.9 (4.8)	95.2 (3.1)	0.335	− 1.3	(− 3.43, 0.83)
	Δ Talar inclination	− 5.5 (5.4)	− 4.8 (3.2)	0.659	− 0.7	(− 2.92, 1.52)
	Tibiotalar tilt angle	1.4 (1.9)	1.9 (1.5)	0.438	− 0.5	(− 1.4, 0.4)
	Δ Tibiotalar tilt angle	− 1.5 (1.3)	− 0.6 (1.7)	0.133	− 0.9	(− 1.7, − 0.1)
II FA n = 13 MA n = 8	Preoperative					
	HKA	6.0 (3.4)	5.4 (3.6)	0.694	0.6	(− 1.25, 2.45)
	LDFA	87.2 (1.5)	86.8 (1.5)	0.549	0.4	(− 0.39, 1.19)
	MPTA	86.5 (1.5)	85.8 (0.9)	0.242	0.7	(0.05, 1.35)
	KJLO	3.2 (1.2)	2.5 (1.2)	0.199	0.7	(0.07, 1.33)
	Tibial plafond inclination	92.9 (5.8)	93.8 (4.0)	0.704	− 0.9	(− 3.53, 1.73)
	Talar inclination	94.9 (5.2)	94.6 (5.1)	0.898	0.3	(− 2.42, 3.02)
	Tibiotalar tilt angle	1.9 (1.6)	1.3 (0.7)	0.342	0.6	(− 0.05, 1.25)
	Postoperative					
	HKA	2.1 (1.4)	1.6 (1.5)	0.490	0.5	(− 0.27, 1.27)
	LDFA	90.2 (3.2)	90.1 (1.7)	0.982	0.1	(− 1.25, 1.45)
	MPTA	89.2 (1.8)	90.1 (1.6)	0.266	− 0.9	(− 1.8, − 0.0)
	KJLO	2.9 (1.6)	3.9 (1.8)	0.215	− 1.0	(− 1.9, − 0.1)
	Δ KJLO	− 0.3 (2.3)	1.4 (1.8)	0.088	− 1.7	(− 2.79, − 0.61)
	Tibial plafond inclination	90.2 (3.0)	90.8 (2.7)	0.648	− 0.6	(− 2.11, 0.91)
	Δ Tibial plafond inclination	− 2.7 (3.6)	− 3.0 (3.9)	0.854	0.3	(− 1.54, 2.14)
	Talar inclination	91.1 (3.0)	89.3 (3.5)	0.219	1.8	(0.08, 3.52)
	Δ Talar inclination	− 3.8 (3.1)	− 5.4 (5.0)	0.391	1.6	(− 0.6, 3.8)
	Tibiotalar tilt angle	0.9 (0.9)	0.5 (0.8)	0.376	0.4	(− 0.05, 0.85)
	Δ Tibiotalar tilt angle	− 1.0 (1.3)	− 0.8 (1.2)	0.660	− 0.2	(− 0.86, 0.46)

Comparing radiological outcomes of FA-TKA and MA-TKA within the CPAK phenotype (Table 5). In the MA-TKA group**,** postoperative KJLO in CPAK 1 was higher than CPAK II (5.1**°** vs. 3.8**°,** *P* = 0.11) but not in the FA-TKA group (3.1° vs. 2.9°, *P*= 0.83), although they did not statistically significant (*P* = 0.11).

**Table 5:** Radiological outcomes of FA-TKA and MA-TKA within CPAK phenotype

CPAK	Radiographic parameters (˚)	CPAK1	CPAK2	p-value	Mean diff	95%CI
		Mean (SD)	Mean (SD)			
MA-TKA CPAK1 = 20 CPAK2 = 8	Preoperative					
	HKA	13.45(5.3)	5.3 (3.6)	< 0.001	8.1	(4.6,11.5)
	LDFA	89.2(2.0)	86.75(1.5)	< 0.001	2.5	(1.1,3.8)
	MPTA	82.8(1.9)	85.7(0.8)	< 0.001	− 3.0	(− 4.0,− 1.9)
	KJLO	3.15(1.3)	2.5(1.2)	0.23	0.7	(− 0.4,1.7)
	Tibial plafond inclination	98.5(4.5)	93.75(4.0)	0.02	4.8	(1.34,8.15)
	Talar inclination	100(4.0)	94.625(5.1)	0.02	5.4	(1.44,9.30)
	Tibiotalar tilt angle	2.45(2.3)	1.25(0.7)	0.05	1.2	(0.05,2.34)
	Postoperative					
	HKA	2.9(2.1)	1.6 (1.5)	0.09	1.3	(− 0.1,2.6)
	LDFA	90.8(2.8)	90.1(1.7)	0.45	0.7	(− 1.06,2.41)
	MPTA	89.45(1.4)	90.1(1.6)	0.33	− 0.7	(− 1.98,0.63)
	KJLO	5.1(1.3)	3.8(1.80)	0.11	1.2	(− 0.16,2.6)
	Tibial plafond inclination	93.4(3.0)	90.7(2.6)	0.03	2.7	(0.43,4.96)
	Talar inclination	95.2(3.1)	89.2(3.5)	< 0.001	6.0	(3.14,8.75)
	Tibiotalar tilt angle	1.85(1.4)	0.5(0.7)	< 0.001	1.4	(0.522,2.17)
FA	Preoperative					
	HKA	11.4 (5.9)	6(3.3)	0.01	5.38	(1.66,9.10)
	LDFA	89.5 (1.9)	87.1 (1.4)	< 0.001	2.38	(1.06,3.70)
	MPTA	82.3 (1.7)	86.4 (1.5)	< 0.001	− 4.08	(− 5.31,− 2.83)
	KJLO	3.6 (1.5)	3.2 (1.2)	0.48	0.38	(− 0.67,1.44)
	Tibial plafond inclination	96.7 (4.1)	92.8 (5.8)	0.06	3.92	(0.06,7.77)
	Talar inclination	99.3(5.1)	94.9 (5.1)	0.04	4.38	(0.42,8.34)
	Tibiotalar tilt angle	2.8 (2.6)	1.8 (1.6)	0.25	1.00	(− 0.67,2.67)
	Postoperative					
	HKA	2.7 (2.7)	2.1 (1.3)	0.43	0.69	(− 0.97,2.36)
	LDFA	89.2 (2.6)	90.15 (3.2)	0.43	− 0.92	(− 3.15,1.30)
	MPTA	88.4(2.6)	89.2 (1.7)	0.34	− 0.85	(− 2.56,0.86)
	KJLO	3.1 (2.0)	2.9 (1.5)	0.83	0.15	(− 1.23,1.53)
	Tibial plafond inclination	91(2.9)	90.1 (2.9)	0.47	0.85	(− 1.43,3.12)
	Talar inclination	93.8 (4.8)	91.8 (2.9)	0.09	2.77	(− 0.32,5.86)
	Tibiotalar tilt angle	1.4(1.9)	0.8 (0.9)	0.38	0.54	(− 0.62,1.69)

### Clinical outcomes

There was no significant difference between mean postoperative VAS pain scores during the first three days after the operation in the FA and MA group (4.3 versus 4.6, *P* = 0.555) (Table 6). At 1 month postoperatively, the FA group had significantly greater knee flexion ROM (110.1 versus 104.5, *P* = 0.042) and achieved significantly higher FJS at 3 months (53.3 versus 46.0, *P* = 0.015) and 6 months (67.8 versus 57.8, *P* < 0.001). Patient satisfaction score at the last follow-up was also higher in the FA group (84.3 versus 79.2, *P* = 0.001).

**Table 6:** Clinical outcomes

Clinical outcomes		FA		MA	Mean Diff	95%CI	Effect Size (Cohen’s d)	*p*-value
	n	Mean (SD)	n	Mean (SD)				
VAS pain score (first 3 days)	30	4.3 (1.4)	30	4.6 (1.6)	− 0.3	− 1.06,0.46	− 0.20	0.555
Flexion range								
Day 3	30	90.5 (11.0)	30	88.8 (10.0)	1.7	− 3.62, 7.02	0.16	0.599
1 month	30	110.1 (9.7)	30	104.5 (11.2)	5.6	0.30, 10.90	0.53	0.042
3 months	30	119.9 (6.9)	30	117.8 (9.3)	2.1	− 2.04, 6.24	0.26	0.325
6 months	28	126.5 (5.5)	28	125.4 (6.5)	1.1	− 2.05, 4.25	0.18	0.483
KOOS								
1 month	30	63.3 (11.3)	30	58.8 (13.6)	4.5	− 1.83, 10.83	0.36	0.161
3 months	30	70.2 (7.2)	30	68.2 (8.1)	2.0	− 1.88, 5.88	0.26	0.304
6 months	28	72.7 (7.4)	28	70.6 (5.8)	2.1	− 1.38, 5.58	0.32	0.246
FJS								
1 month	30	34.9 (8.9)	30	31.7 (9.3)	3.2	− 1.41, 7.81	0.35	0.179
3 months	30	53.3 (9.5)	30	46.0 (12.2)	7.3	1.77, 12.83	0.67	0.015
6 months	28	67.8 (9.5)	28	57.8 (10.2)	10.0	4.84, 15.16	1.01	< 0.001
Patient satisfaction score	30	84.3 (6.1)	30	79.2 (5.3)	5.1	0.21, 7.99	0.89	0.001

With regard to CPAK phenotypes (Table 7), in knees with CPAK type I, the FA group had significantly better FJS scores at all time points (38.0 versus 31.0, *P* = 0.040 at 1 month; 56.9 versus 45.0, *P* = 0.002 at 2 months; and 72.9 versus 57.3, *P* < 0.001 at 6 months), and they also had better satisfaction scores (85.8 versus 78.5, *P* = 0.029).

**Table 7:** Clinical outcomes CPAK type 1 and 2 subgroups

CPAK	Clinical outcomes		FA		MA	p-value	Mean diff	95%CI
		n	Mean (SD)	n	Mean (SD)			
I	VAS pain score (first 3 days)	13	4.5 (1.2)	20	4.2 (1.8)	0.590	0.3	(− 0.84, 1.44)
	Flexion range							
	Preoperative	13	120.2 (9.7)	20	120.6 (7.5)	0.896	− 0.4	(− 7.31, 6.51)
	Day 3	13	89.0 (8.2)	20	87.0 (11.5)	0.569	2.0	(− 5.48, 9.48)
	1 month	13	105.9(11.0)	20	102.8(12.0)	0.451	3.1	(− 5.75, 11.95)
	3 months	13	118.0(8.4)	20	116.7(9.7)	0.711	1.3	(− 5.64, 8.24)
	6 months	12	125.1 (5.6)	18	124.1 (6.3)	0.669	1.0	(− 3.57, 5.57)
	KOOS							
	Preoperative	13	33.8 (11.5)	20	35.6 (16.5)	0.736	− 1.8	(− 12.43, 8.83)
	1 month	13	66.0 (11.3)	20	59.0 (13.0)	0.123	7.0	(− 2.31, 16.31)
	3 months	13	70.0 (5.9)	20	68.5 (7.8)	0.570	1.5	(− 3.71, 6.71)
	6 months	12	73.4 (6.6)	18	70.3 (5.9)	0.190	3.1	(− 1.82, 8.02)
	FJS							
	Preoperative	13	25.3 (17.8)	20	24.8 (16.3)	0.931	0.5	(− 12.87, 13.87)
	1 month	13	38.0 (9.3)	20	31.0 (8.9)	0.040	7.0	(− 0.1, 14.1)
	3 months	13	56.9 (9.0)	20	45.0 (10.8)	0.002	11.9	(4.33, 19.47)
	6 months	12	72.9 (7.2)	18	57.3 (8.9)	< 0.001	15.6	(9.46, 21.74)
	Patient satisfaction score	13	85.8 (6.0)	20	78.5 (4.3)	< 0.001	7.3	(3.11, 11.49)
II	VAS pain score (first 3 days)	13	4.0(1.7)	8	5.5(0.8)	0.029	− 1.5	(− 2.8, − 0.2)
	Flexion range							
	Preoperative	13	122.5 (10.8)	8	126.3 (5.8)	0.374	− 3.8	(− 12.38, 4.78)
	Day 3	13	92.7(10.8)	8	93.0 (4.6)	0.940	− 0.3	(− 8.36, 7.76)
	1 month	13	113.2 (6.4)	8	109.3 (8.7)	0.249	3.9	(− 4.5, 12.3)
	3 months	13	120.8 (5.8)	8	122.4 (5.3)	0.535	− 1.6	(− 7.44, 4.24)
	6 months	12	127.5 (5.7)	8	129.0 (4.8)	0.546	− 1.5	(− 6.98, 3.98)
	KOOS							
	Preoperative	13	39.3 (12.9)	8	34.1 (13.3)	0.392	5.2	(− 8.77, 19.17)
	1 month	13	62.0 (12.2)	8	58.5 (12.4)	0.526	3.5	(− 9.6, 16.6)
	3 months	13	69.0 (7.9)	8	67.8 (9.0)	0.756	1.2	(− 7.94, 10.34)
	6 months	12	70.0 (6.6)	8	71.2 (4.0)	0.659	− 1.2	(− 6.67, 4.27)
	FJS							
	Preoperative	13	30.3 (15.9)	8	27.1 (14.6)	0.649	3.2	(− 12.85, 19.25)
	1 month	13	32.9 (8.3)	8	32.6 (9.4)	0.940	0.3	(− 9.26, 9.86)
	3 months	13	49.2 (9.8)	8	48.2 (12.0)	0.833	1.0	(− 10.91, 12.91)
	6 months	12	61.3 (8.5)	8	58.9 (11.1)	0.586	2.4	(− 8.43, 13.23)
	Patient satisfaction score	13	82.3 (6.7)	8	80.6 (5.6)	0.559	1.7	(− 4.72, 8.12)

Comparing clinical outcomes of FA-TKA and MA-TKA within CPAK phenotype (Table 8), FA-TKA in the CPAK I group demonstrated significantly better Forgotten Joint Scores (FJS) at 6 months compared to CPAK II (72.9 ± 7.2 vs. 61.2 ± 8.4, *P* = 0.002).Additionally, MA-TKA in the CPAK II group had significantly higher VAS pain scores in the first three days (5.5 ± 0.75 vs. 3.8 ± 2.1, *P* = 0.042).

**Table 8:** Clinical outcomes of FA-TKA and MA-TKA within CPAK phenotype

	Clinical outcomes	CPAK I		CPAK II		p-value	Mean Diff	95%CI
		n	Mean (SD)	n	Mean (SD)			
FA-TKA	VAS pain score (first 3 days)	13	4.15 (1.7)	13	3.77 (2.0)	0.605	0.38	− 1.1,1.8
	Flexion range							
	Preoperative	13	120.2 (9.7)	13	122.6 (10.5)	0.572	− 2.3	− 10.6,5.9
	Day 3	13	89.0 (8.2)	13	92.0 (10.8)	0.348	− 3.6	− 11.4,4.1
	1 month	13	105.9(11.0)	13	113.1(6.3)	0.052	− 7.2	− 14.5,0.07
	3 months	12	117.0(8.2)	13	120.77(5.8)	0.219	− 3.6	− 9.4,2.2
	6 months	12	125.0 (5.6)	12	127.5 (5.6)	0.306	− 2.4	− 7.1,2.3
	KOOS							
	Preoperative	13	33.8 (11.5)	13	39.2 (12.8)	0.265	4.7	− 15.3,4.4
	1 month	13	66.0 (11.2)	13	62.0 (12.1)	0.396	4.5	− 5.5,13.4
	3 months	13	70.0 (5.9)	13	68.9 (7.8)	0.723	2.7	− 4.6,6.6
	6 months	11	73.6 (6.8)	12	70.0 (6.5)	0.216	2.8	− 2.2,9.4
	FJS							
	Preoperative	13	25.3 (17.8)	13	30.2 (15.8)	0.460	6.6	− 18.6,8.6
	1 month	13	38.0 (9.3)	13	32.8 (8.3)	0.152	3.4	− 2.0,12.2
	3 months	13	56.9 (8.9)	13	49.1 (9.7)	0.047	3.6	0.10,15.2
	6 months	12	72.9 (7.2)	12	61.2 (8.4)	0.002	3.2	4.9,18.3
	Patient satisfaction score	13	85.8 (6.0)	13	82.3 (6.6)	0.178	3.4	− 1.6,8.6
MA-TKA	VAS pain score (first 3 days)	20	3.8(2.1)	8	5.5(0.75)	0.042	− 1.6	− 3.2,1.5
	Flexion range							
	Preoperative	20	120.5(7.4)	8	126.3 (5.8)	0.065	− 5.7	− 11.7,0.3
	Day 3	20	86.9(11.5)	8	93.0 (4.6)	0.166	− 6.0	− 14.7,2.6
	1 month	20	102.7(12.0)	8	109.3 (8.7)	0.178	− 6.5	− 16.1,3.1
	3 months	19	16.1(9.6)	8	122.4 (5.3)	0.100	− 6.2	− 13.7,1.2
	6 months	18	124.1(6.3)	8	129.0 (4.8)	0.063	− 4.8	− 10.0,0.2
	KOOS							
	Preoperative	20	35.5(16.4)	8	34.1 (13.3)	0.827	1.4	− 12.0,14.9
	1 month	20	59.0(13.0)	8	58.5 (12.4)	0.921	0.5	− 10.5,11.6
	3 months	20	68.4(7.8)	8	67.8 (9.0)	0.839	0.7	− 6.3,7.6
	6 months	17	70.0 (6.0)	8	71.2 (4.0)	0.738	− 0.8	− 5.6,4.0
	FJS							
	Preoperative	20	24.7(16.2)	8	27.1 (14.6)	0.733	− 2.2	− 15.9,11.3
	1 month	20	31.0(8.9)	8	32.6 (9.4)	0.693	− 1.5	− 9.2,6.2
	3 months	20	45.0(10.8)	8	48.2 (12.0)	0.501	− 3.1	− 12.7,6.3
	6 months	18	57.2(8.9)	8	58.9 (11.1)	0.705	− 1.5	− 9.9,6.8
	Patient satisfaction score	20	78.5(4.3)	8	80.6 (5.6)	0.291	− 2.1	− 6.1,1.9

## Discussion

This prospective RCT of bilateral TKA aimed to compare the effects of different TKA modalities, MA-TKA and FA-TKA, on the same patient. The results of this trial demonstrate that while overall alignment was comparable, since FA-TKA aims to restore the pre-arthritic knee joint surface, considering the joint configuration, menisci, and the function of the collateral and cruciate ligaments, which are key determinants of normal knee kinematics. This approach contrasts with the mechanical alignment strategy, which primarily focuses on the relative position of the hip, knee, and ankle joints, as seen in MA-TKA. Resulting in less soft tissue release. FA patients had balance in 66.7% of knees, which was almost triple that achieved in the MA group (23.3%).

Masilamani et al. [12] compared balance achieved with FA versus MA in bilateral TKA and found that the FA group achieved balance in 66.2% of knees compared to 32.3% in the MA group, similar to our findings.

The present study showed postoperative KJLO in FA-TKA was reduced and more parallel to the floor, while in the MA-TKA it increased, slanted down to the lateral side, and was less parallel to the floor.

Ji et al. [5] reported similar results when performing KA-TKA. Their study involved 3 groups undergoing conventional MA-TKA, navigated MA-TKA, and KA-TKA, with 65 knees in each group. Postoperative KJLO in conventional MA-TKA and navigated MA-TKA increased and slanted down to the lateral side (from 2.5° to 3.3° and from 2.3° to 2.6°, respectively). In the KA-TKA group, postoperative KJLO decreased and was more parallel to the floor (from 1.7° to 0.6°).

Regarding to impact of KJLO and longevity of prothesis, BAE, K. et al. [17] reported that base on phenotypes based on the combined assessment of the hip-knee-ankle (HKA) angle and JLO, measured on standing radiographs only varus alignment-lateral joint-line inclination shows statistical significant decrease in longevity in 10 and 15 years compared to control group (neutral alinment-parallel joint line) (from 97 to 93% vs. from 90 to 69%; *P* = 0.017, < 0.001). Compared to our study, there should not be any difference in the longevity of prothesis between the two groups.

Victor et al. [4] studied 248 young healthy individuals and 532 patients with knee arthritis and found that tibial (or knee in this study) joint line angle was parallel to the floor in healthy individuals with neutral alignment (mean 0.3°, SD 1.9), and even in patients with constitutional varus, tibial joint line angle was parallel to the floor (mean 0.3°, SD 1.9). In patients with symptomatic varus knee arthritis, the tibial joint line slanted down to the lateral side and was less parallel to the floor [4, 5].

KJLO is a dynamic parameter and can be affected by many factors. Lee et.al discovered that it was strongly influenced by the distance between the feet when taking full-limb radiography and that it was also affected by the combined impacts of LDFA, MPTA, and ankle joint line orientation [18]. Toyono et al. [19] found that performing standing long leg radiographs, open (both feet apart at shoulder width) and closed stance (feet in contact) resulted in differences in KJLO, ankle joint line orientation, and lower limb mechanical axis. When assessing the KJLO and/or ankle joint line orientation, it is therefore important to take into consideration the distance between the feet, which should be standardized. While the ideal distance between the feet for the assessment of KJLO and ankle joint line has not yet been definitively established, Krackow reported that the closed stance had the most similar foot position to that when walking [20].

In our study, all three ankle parameters, including tibial plafond inclination, talar inclination, and tibiotalar tilt ankle improved after performing TKA, without significant differences between the FA and the MA group.

Previous studies have reported that tibial plafond inclination and talar inclination improved after TKA [21–23]. In contrast, tibiotalar tilt angle was not significantly altered after TKA [21] and can worsen with an increase in the degree of varus ankle incongruency when knee varus deformity is ≥ 10° [22] or when varus correction is ≥ 10° [21]. With regard to tibiotalar tilt angle, their results were different from those of our study, in which tibiotalar tilt angle improved after TKA. The reason for this difference may be because our study was performed in bilateral TKA, so that knee alignment improved in both limbs, and when weight-bearing radiographs were taken, the effects on ankle alignment may therefore have been different compared to those of the other studies of unilateral TKA. Another reason was the radiographic assessment; while our RCT employed closed-leg standing long leg radiographs, the other studies used open-leg radiographs and may not have fully clarified the feet positioning. Further research is needed to address this issue.

The FA group in this study, in which the initial plan was based on kinematic alignment concepts, achieved significantly higher FJS at 3 and 6 months, as well as higher patient satisfaction scores. A recent study by Jeffrey et al. [24] found that FA-TKA had better FJS compared to adjusted MA-TKA. Clark et al. [25] also reported similar results, revealing that FA-TKA with an initial kinematic alignment plan [FA(k)] had significantly better FJS than FA-TKA with an initial mechanical alignment plan [FA(m)].

More than half (55%) of the knees in our study had preoperative constitutional varus with apex distal joint line orientation or CPAK type I, followed by 32% with CPAK type II (neutral aHKA and apex distal joint line orientation). This differs from the results of other studies [8, 25, 26], which had only around 19.4%–30% with preoperative CPAK type I. However, a recent study of Japanese patients with knee osteoarthritis found a similar distribution to that of ours, with 53.8% CPAK type I followed by 25.4% with CPAK type II. The reasons behind these variations may be related to ethnicity and a more varus MPTA [27].

With regard to radiographic and clinical outcomes in different CPAK phenotypes, the effects of FA-TKA on KJLO and FJS on CPAK type I were significantly different from those of MA-TKA. A knee with constitutional varus results in a more varus proximal tibial angle, with the KJLO more parallel to the floor; in contrast, a more valgus or even perpendicular knee produces a KJLO slanted more to the lateral side and ends up less parallel to the floor. When looking at the clinical outcomes, FA-TKA in CPAK type I gave better FJS at all measurement time points, including 1, 3, and 6 months postoperatively, and achieved higher patient satisfaction scores.

### Limitation

This study had some limitations that should be considered. First of all, it had a small sample size, although our study does provide enough power for detecting differences between groups. The study was inadequately powered to assess outcomes in other knee phenotypes. Second, this was a short-term study, which precludes us from assessing the clinical outcomes, safety, and longevity of the implant, as well as ankle symptoms that may develop several years after TKA [28]. However, our studies do show that FA-TKA results in a joint line orientation more horizontal and closer to the native ankle joint compared to MA-TKA, leading to clinical and biomechanical advantages, but these do not necessarily imply superior prosthesis longevity or survival. Other factors, such as tibial component orientation in the sagittal plane [29] and the maintenance of parallelism during the gait cycle [30], also play a role in implant survival. Eventually, a long-term follow-up is still needed to reach a conclusion. Third, this study focused only on ankle radiographic change; subtalar joint compensation and hindfoot alignment were not evaluated. Fourth, our study is a single-center design that consists of mostly Asian patients. Despite this, our data show some similarity with other Asian populations [27]. Future multicenter studies are warranted to validate our findings and enhance the generalizability and external validity of the results. Fifth, although the surgeon in our study was specialized in doing robotic surgery using both MA-TKA and FA-TKA techniques, it is worth noting that robotic TKA surgery requires a learning curve of approximately 30 cases to reach the proficiency stage, which results in reduced operative time and improved clinical outcome [31]. Finally, the risk and benefit between unilateral and bilateral TKA are still controversial, although bilateral TKA does offer.

## Conclusion

FA-TKA can correct overall lower limb alignment and improve ankle joint line similarly to MA-TKA, but with less soft tissue release required. In patients with constitutional varus and apex distal joint line orientation (CPAK type I), FA-TKA is more beneficial and can result in KJLO being more parallel to the floor, with higher FJS and greater patient satisfaction compared to MA-TKA.

## Data Availability

The authors confirmed that raw data and supplementary data of this study will be provided upon reasonable request.

## References

[1] Ritter MA. The Anatomical Graduated Component total knee replacement: A long-term evaluation with 20-year survival analysis. The Journal of Bone and Joint Surgery British volume. 2009 Jun;91-B(6):745–9. 10.1302/0301-620X.91B6.21854 19483226

[2] Bourne RB, Chesworth BM, Davis AM, Mahomed NN, Charron KDJ. Patient Satisfaction after Total Knee Arthroplasty: Who is Satisfied and Who is Not? Clin Orthop Relat Res. 2010;468(1):57–63. 19844772 10.1007/s11999-009-1119-9 PMC2795819

[3] Gunaratne R, Pratt DN, Banda J, Fick DP, Khan RJK, Robertson BW. Patient Dissatisfaction Following Total Knee Arthroplasty: A Systematic Review of the Literature. J Arthroplasty. 2017;32(12):3854–60. 28844632 10.1016/j.arth.2017.07.021

[4] Victor JMK, Bassens D, Bellemans J, Gürsu S, Dhollander AAM, Verdonk PCM. Constitutional Varus Does Not Affect Joint Line Orientation in the Coronal Plane. Clin Orthop Relat Res. 2014;472(1):98–104. 23733590 10.1007/s11999-013-2898-6 PMC3889437

[5] Ji HM, Han J, Jin DS, Seo H, Won YY. Kinematically aligned TKA can align knee joint line to horizontal. Knee Surg Sports Traumatol Arthrosc. 2016;24(8):2436–41. 26811035 10.1007/s00167-016-3995-3

[6] Kim JT, Han J, Lim S, Shen QH, Won YY. Kinematically Aligned TKA Aligns the Ankle Joint Line Closer to Those of the Native Ankle than Mechanically Aligned TKA in Bipedal Stance. J Knee Surg. 2019;32(10):1033–8. 31434142 10.1055/s-0039-1694796

[7] Kayani B, Konan S, Tahmassebi J, Oussedik S, Moriarty PD, Haddad FS. A prospective double-blinded randomised control trial comparing robotic arm-assisted functionally aligned total knee arthroplasty versus robotic arm-assisted mechanically aligned total knee arthroplasty. Trials. 2020;21(1):194. 32070406 10.1186/s13063-020-4123-8 PMC7027302

[8] MacDessi SJ, Griffiths-Jones W, Harris IA, Bellemans J, Chen DB. Coronal Plane Alignment of the Knee (CPAK) classification: a new system for describing knee phenotypes. The Bone & Joint Journal. 2021 Feb 1;103-B(2):329–37. 10.1302/0301-620X.103B2.BJJ-2020-1050.R1 PMC7954147 33517740

[9] Behrend H, Giesinger K, Giesinger JM, Kuster MS. The “Forgotten Joint” as the Ultimate Goal in Joint Arthroplasty. J Arthroplasty. 2012;27(3):430-436.e1. 22000572 10.1016/j.arth.2011.06.035

[10] Roos EM, Roos HP, Lohmander LS, Ekdahl C, Beynnon BD. Knee Injury and Osteoarthritis Outcome Score (KOOS)—Development of a Self-Administered Outcome Measure. J Orthop Sports Phys Ther. 1998;28(2):88–96. 9699158 10.2519/jospt.1998.28.2.88

[11] Tang, Q., Yu, Hc., Shang, P. et al. Selective medial soft tissue release combined with tibial reduction osteotomy in total knee arthroplasty. J Orthop Surg Res 12, 174 (2017). 10.1186/s13018-017-0681-1 10.1186/s13018-017-0681-1 PMC5686887 29137667

[12] Howell SM, Papadopoulos S, Kuznik KT, Hull ML. Accurate alignment and high function after kinematically aligned TKA performed with generic instruments. Knee Surg Sports Traumatol Arthrosc. 2013;21(10):2271–80. 23948721 10.1007/s00167-013-2621-x

[13] Lee JH, Jeong BO. Radiologic Changes of Ankle Joint after Total Knee Arthroplasty. Foot Ankle Int. 2012;33(12):1087–92. 23199858 10.3113/FAI.2012.1087

[14] Dossett HG, Swartz GJ, Estrada NA, LeFevre GW, Kwasman BG. Kinematically Versus Mechanically Aligned Total Knee Arthroplasty. Orthopedics. 2012;35(2):e160–9. 22310400 10.3928/01477447-20120123-04

[15] Nishimoto J, Tanaka S, Inoue Y, Tanaka R. Minimal clinically important differences in short-term postoperative Knee injury and Osteoarthritis Outcome Score (KOOS) after total knee arthroplasty: A prospective cohort study. Journal of Orthopaedics, Trauma and Rehabilitation. 2024;31(1):15–20. 10.1177/22104917231181644.

[16] Fujimoto E, Sasashige Y, Tomita T, Kashiwagi K, Inoue A, Sawa M, Ota Y. Different femorotibial contact on the weight-bearing: midflexion between normal and varus aligned knees after total knee arthroplasty. Knee Surg Sports Traumatol Arthrosc. 2015;23:1720–8. 25059339 10.1007/s00167-014-3194-z

[17] BAE, K. et al. Effect of joint-line obliquity on long-term survivorship of total knee arthroplasty: A postoperative phenotype analysis. Knee Surgery, Sports Traumatology, Arthroscopy, *[s. l.]*, v. 32, n. 12, p. 3230–3238, 2024. 10.1002/ksa.12311. Disponível em: https://research.ebsco.com/linkprocessor/plink?id=5d9d7aa3-e2b0-3484-b276-429bcb7ab2e3. Acesso em: 2 mar. 2025. 10.1002/ksa.12311 38895851

[18] Masilamani ABS, Jayakumar T, Mulpur P, Gandhi V, Kikkuri RR, Reddy AVG. Functional alignment is associated with increased incidence of pre-balance, reduced soft-tissue release, and post-operative pain compared to mechanical alignment in patients undergoing simultaneous bilateral robotic-assisted TKA. J Robotic Surg. 2023;17(6):2919–27. 10.1007/s11701-023-01732-6 37831402

[19] Lee NK, Kim TW, Lee S, Choi YS, Kang SB, Chang CB. Effect of distance between the feet on knee joint line orientation after total knee arthroplasty in standing full-limb radiographs. Knee Surg Sports Traumatol Arthrosc. 2022;30(9):3032–40. 34269849 10.1007/s00167-021-06662-0

[20] Toyono S, Suzuki A, Nakajima T, Wanezaki Y, Aso M, Yamamoto T, et al. Knee joint line orientation after total knee arthroplasty is affected by the mechanical axis inclination of the lower limb according to foot position. Journal of Joint Surgery and Research. 2023;1(1):123–7.

[21] Krackow KA. Approaches to planning lower extremity alignment for total knee arthroplasty and osteotomy about the knee. Adv Orthop Surg. 1983;7:69–88.

[22] Jin G, Fan Y, Jiang L, Chen Z, Wang C. MAKO robot-assisted total knee arthroplasty cannot reduce the aggravation of ankle varus incongruence after genu varus correction ≥ 10°: a radiographic assessment. BMC Musculoskelet Disord. 2023;24(1):492. 37322501 10.1186/s12891-023-06597-2 PMC10268520

[23] Chang CB, Chung CY, Park MS, Choi JH, Kim JS, Lee KM. Aggravation of Ankle Varus Incongruency Following Total Knee Replacement Correcting ≥10° of Genu Varum Deformity: A Radiographic Assessment. J Arthroplasty. 2020;35(11):3305–10. 32646678 10.1016/j.arth.2020.06.027

[24] Gursu S, Sofu H, Verdonk P, Sahin V. Effects of total knee arthroplasty on ankle alignment in patients with varus gonarthrosis: Do we sacrifice ankle to the knee? Knee Surg Sports Traumatol Arthrosc. 2016;24(8):2470–5. 26590564 10.1007/s00167-015-3883-2

[25] Jeffrey M, Marchand P, Kouyoumdjian P, Coulomb R. Short-term functional outcomes of robotic-assisted TKA are better with functional alignment compared to adjusted mechanical alignment. SICOT-J. 2024;10:2. 38240728 10.1051/sicotj/2024002 PMC10798231

[26] Clark GW, Steer RA, Khan RN, Collopy DM, Wood D. Maintaining Joint Line Obliquity Optimizes Outcomes of Functional Alignment in Total Knee Arthroplasty in Patients With Constitutionally Varus Knees. J Arthroplasty. 2023;38(7):S239–44. 37061140 10.1016/j.arth.2023.04.004

[27] Karasavvidis T, Pagan CA, Debbi EM, Mayman DJ, Jerabek SA, Vigdorchik JM. No Difference in Limb Alignment Between Kinematic and Mechanical Alignment Robotic-Assisted Total Knee Arthroplasty. J Arthroplasty. 2024;39(8):S200–5. 38548234 10.1016/j.arth.2024.03.050

[28] Toyooka S, Osaki Y, Masuda H, Arai N, Miyamoto W, Ando S, et al. Distribution of Coronal Plane Alignment of the Knee Classification in Patients with Knee Osteoarthritis in Japan. J Knee Surg. 2023;36(07):738–43. 35114721 10.1055/s-0042-1742645

[29] Bernard R. Fundamentals of biostatistics. 5thed: Duxbury Press; 2000

[30] Kastner N, Sternbauer S, Friesenbichler J, Vielgut I, Wolf M, Glehr M, Leithner A, Sadoghi P. Impact of the tibial slope on range of motion after low-contact-stress, mobile-bearing, total knee arthroplasty. Int Orthop. 2014;38:291–5. 24346515 10.1007/s00264-013-2242-5 PMC3923942

[31] Leandro Ejnisman, Eliane Antonioli, Luciana Cintra, Pamela Gabriela de Oliveira Souza, Lauro Augusto Veloso Costa, Mario Lenza,Robot-assisted knee arthroplasty: Analyzing the learning curve and initial institutional experience,Computational and Structural Biotechnology Journal,Volume 24,2024 10.1016/j.csbj.2024.04.013 PMC11068478 38706810

